# The effectiveness of community-based cycling promotion: findings from the *Cycling Connecting Communities *project in Sydney, Australia

**DOI:** 10.1186/1479-5868-7-8

**Published:** 2010-01-27

**Authors:** Chris E Rissel, Carolyn New, Li Ming Wen, Dafna Merom, Adrian E Bauman, Jan Garrard

**Affiliations:** 1 Health Promotion Service, Sydney South West Area Health Service, Hugh Jardine Building, Eastern Campus, Liverpool Hospital, Locked Mail Bag 7017, Liverpool BC 1871, Australia; 2 Sydney Medical School, K25 - Medical Foundation Building, The University of Sydney, NSW 2006 Australia; 3 School of Health & Social Development, Deakin University, Burwood Highway, Burwood Victoria 3125, Australia

## Abstract

**Background:**

Encouraging cycling is an important way to increase physical activity in the community. The Cycling Connecting Communities (CCC) Project is a community-based cycling promotion program that included a range of community engagement and social marketing activities, such as organised bike rides and events, cycling skills courses, the distribution of cycling maps of the area and coverage in the local press. The aim of the study was to assess the effectiveness of this program designed to encourage the use of newly completed off-road cycle paths through south west Sydney, Australia.

**Methods:**

The evaluation used a quasi-experimental design that consisted of a pre- and post-intervention telephone survey (24 months apart) of a cohort of residents (n = 909) in the intervention area (n = 520) (Fairfield and Liverpool) and a socio-demographically similar comparison area (n = 389) (Bankstown). Both areas had similar bicycle infrastructure. Four bicycle counters were placed on the main bicycle paths in the intervention and comparison areas to monitor daily bicycle use before and after the intervention.

**Results:**

The telephone survey results showed significantly greater awareness of the Cycling Connecting Communities project (13.5% vs 8.0%, p < 0.05) in the intervention area, with significantly higher rates of cycling in the intervention area (32.9%) compared with the comparison area (9.7%) amongst those aware of the project. There was a significant increase in use of bicycle paths in the intervention area (28.3% versus 16.2%, p < 0.05). These findings were confirmed by the bike count data.

**Conclusion:**

Despite relatively modest resources, the Cycling Connecting Communities project achieved significant increases in bicycle path use, and increased cycling in some sub-groups. However, this community based intervention with limited funding had very limited reach into the community and did not increase population cycling levels.

## Background

Riding a bicycle has considerable health benefits, with longitudinal studies reporting 30-40% decreases in mortality for regular riders [1,2] and decreased risk of diabetes [3]. Health benefits from commuter cycling include less likelihood of being overweight or obese [4], and considerable savings (estimated at $237 (AUD) million per annum) to the health budget [5]. Cycling for transport also has benefits for the environment, producing zero carbon emissions, contributes to less traffic congestion, and results in lower exposure of the rider to traffic pollution [4,6].

Despite cycling being the third most popular recreational activity in Australia [7], the proportion of trips by bicycle in Australia is about one per cent [8], similar to New Zealand and the USA, but far lower than in many European cities [9]. Although often poorly evaluated, Australian interventions to increase levels of cycling have generally been successful within the populations studied [10].

There has been very little Australian or international research evaluating the effectiveness of infrastructure and environmental changes upon increasing population levels of physical activity [11]. One example that building and promoting adequate cycleway facilities increases regular cycling comes from Bikewest in the Western Australian Department of Transport. They have used the mass marketing message *Cycle Instead*, complemented by an individualised marketing program conducted by a 'Travelsmart' team from the same Department, and reported a 53% increase in bike trips at 12 month follow-up [12].

A new Sydney Roads and Traffic Authority (RTA) built shared pedestrian and bicycle path, the Parramatta-Liverpool Rail-Trail was recently evaluated [13], one of the few such studies internationally. With only minimal promotion of the Rail-Trail, moderate increases in trail use and small increases in cycling activity among residents who live within 1.5 kms to trail were found [13]. However, there was no control area/trail and those increases that were observed may have been due to general increases in cycling in NSW [14].

It is unknown if promotion of bicycle paths leads to an increase in the proportion of adults who meet the physical activity recommendation, or whether the new cycle path simply attracts existing cyclists away from other routes and away from other modes of exercise. A prospective study in the US found that the building of a multi-use trail did not demonstrate an increase in physical activity among adults living near the trail [15]. Further, while often suggested, it is not clearly documented that an increase in cycling leads to an increase in population physical activity levels. Therefore, the two research questions of the Cycling Connecting Communities (CCC) project were 1) Does promoting new infrastructure increase cycling? and 2) Would an increase in cycling result in an increase in population levels of physical activity?

## Methods

### The community based intervention

The CCC project interventions were supported by a large number of partners through an Advisory Committee, including representatives from two local government areas (Liverpool and Fairfield City Councils) who supported and promoted CCC activities. Fairfield City Council had already initiated their own cycling related projects consisting of a Bicycle Recycle project to improve access to cheap bikes and the setup of a local bicycle group in the Fairfield area, called the Western Sydney Cycling Network.

The intervention program was based on a social marketing framework applied locally and used behaviour change theories including the transtheoretical model and stages of change [16] (see Table 1).

**Table 1:** Overview of project strategies

Strategies	Activities	When
Media launch		September 2007

Information distribution	Bike map and information leaflet	Ongoing

Skills and proficiency	Free courses	Sessions offered each season

Awareness	Use of local media One hour community and workplace presentations	Ongoing 2008

Trialling - easy level	Community rides	Late 2008 and 2009

Trialling - commuting	Ride to Work Day	October 2007 and 2008

Trialling - intermediate level	Spring Cycle	October 2008

Transport trip generators	Colleges of Technical and Further Education (TAFE)	On-going

The project was implemented in the local government areas of Liverpool and Fairfield, with a third adjoining local government area (Bankstown) as the comparison area. All three areas are characterised by higher levels of non-English speaking residents compared to the rest of Sydney, and higher levels of social disadvantage [17]. Addressing social equity issues was a condition of funding approval.

A range of project resources was produced or purchased and branded with the project name and logo. A map titled *'Discover Fairfield and Liverpool by Bike' *showing the bicycle paths and useful cycling routes in the area was considered the key resource in raising awareness for non and infrequent cyclists by illustrating the extent of local bike paths. 20,000 maps were produced. A general information booklet addressing concerns of potential cyclists titled *'Thinking about cycling' *was created to complement the map (n = 5,000). Water bottles (n = 2,000) and reflective slap bands (n = 2,000) were designed with specific project images to serve as cues to engage in cycling.

As part of the CCC project, a one-hour presentation was developed and delivered to 351 people attending 24 community or workplace groups between February and September 2008. The objective was to raise awareness of cycling, the benefits of physical activity, the CCC project activities and resources, and to generate discussion of how to progress to riding a bike or to riding a bike more.

One of the main interventions in the early stages of CCC was the offer of free cycle skills courses. These courses were designed for members of the public who wanted to ride but did not, and focused on basic skills and confidence [18].

National Ride to Work Day is a national event which is part of a behaviour change program run by Bicycle Victoria to encourage workers to commute to work by bike on that day [19]. The CCC project trialled this as a broader community event in 2007, with a community breakfast held in a park adjacent to a major teaching Hospital in Liverpool. As this was considered successful, the event was replicated in 2008 with a higher level of marketing to local businesses.

### Community rides

A number of community rides were organised, some as part of NSW Bike Week, a state-wide NSW Government initiative. Councils and other organisations are encouraged to run organised bicycle events in a safe and supported environment. The RTA provides start-up funding to assist in the promotion of these events, and rides were organised in each of the intervention area councils each year. Approximately 100 people participated in these rides.

The City of Sydney Spring Cycle is an annual event that is run by Bicycle New South Wales (NSW) [20]. While it has historically run from North Sydney to Olympic Park, additional starts were proposed for 2008. The CCC project lobbied Bicycle NSW to include the Liverpool start in 2008, and this was agreed upon with volunteer support from the CCC project. Several hundred people participated in the inaugural Liverpool start.

#### Australian Better Health Initiative funded community rides

The success of the Liverpool Bike Week event provided a model that could be replicated in other local communities in Liverpool and Fairfield. To make it more accessible to lower socio-economic areas, it was also desirable to provide free bike hire. A grant from the Australian Better Health Initiative (ABHI) provided the opportunity to run four such events over a four month period in 2009.

Four localities were chosen where there was good access to a network of cycle paths. Two were identified in the Liverpool area and two in Fairfield, and each site could be supported by the relevant local bicycle user group. Resources available on the day included a leaflet describing the route, healthy recipe books, and *Measure Up *booklets and measuring tapes, and CCC project resources. Participation varied on these rides, depending on the weather, ranging from 10-100.

#### Budget

The CCC was awarded $292,000 (AUS) from 2007 to 2009, which included evaluation, project coordination and intervention costs.

#### Evaluation

The impact evaluation used two approaches (Study 1 and 2) and two different data sources.

#### Study 1: Research questions related to telephone surveys

1. Is there a significant increase in self-reported cycle path use for cycling or walking, in the percentage of cyclists who used the cycle path in the past month and did this use vary across population sub-groups (age, sex, education attainment, ethnicity, car owners)?

2. Did the intervention campaign result in a significant increase in unprompted and prompted awareness of the cycle path?

3. Did the intervention result in a significant increase in cycling commuting or recreational cycling and who are more likely to change these behaviours?

The evaluation design was quasi-experimental with a cohort study with two data collection points in the intervention and comparison areas. The cohort evaluation focused on a random sample of adults, aged 18 years or older, living within two kilometres from the cycleway in suburbs that were defined as the intervention area or the comparison area, a different but demographically similar part of Sydney adjacent to the intervention area.

### Sample

Respondents were selected using a three-stage sampling process. In the first stage postcodes within two kilometres from the two bicycle paths were identified. In the second sampling stage households in these areas were linked to the Electronic White Page Directory (EWPD) to randomly select telephone numbers for each sample group. In the third stage each household was telephoned and screened for eligible respondents. Eligible respondents were aged 18 years or older, and spoke English. If there was more than one eligible person per household, respondents were selected randomly using the most recent birthday technique.

### Data collection

**Figure 1:**
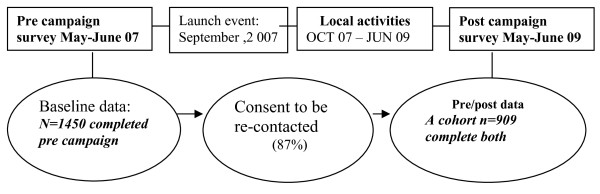
**Design of impact evaluation using a telephone survey**.

Data were collected using standard computer assisted telephone interview techniques (CATI). The baseline interview (approximately 10 minutes) was conducted in May-June 2007. Respondents who consented to participate in a follow-up interview were re-contacted 24 months later, with follow-up interviews conducted in May-June 2009 (see Figure 1). Interviews were conducted using a commercial market research company Socio-demographic characteristics (including age, sex, educational attainment, income, marital status, presence of children in the household and car ownership) were asked only at baseline using questions previously used in the NSW Health Survey [21]. These questions were replaced with campaign process evaluation questions in the follow-up interview.

### Main outcome measures

Frequency of cycling - When was the last time you rode a bicycle? Was it today, in the last week, in the last month, in the last year, longer than a year, or never?

Physical activity (PA) behaviour -

• Sufficiently active: sufficient to confer health benefit if total time is greater or at least 150 minutes (using the Active Australia questionnaire).

• Total time cycling per week: estimated time spent on cycling in the past week.

• Total sessions of cycling per week: number of times spent on cycling continuously for at least 10 minutes in the past week.

Usage of bicycle paths - whether respondent had ever used the new bicycle paths for any purpose.

### Statistical analysis

All data analysis was conducted using STATA [22]. For the cohort of survey respondents for whom there was both baseline and follow-up data, regression analyses (general linear regression was used for continuous measures and logistic regression was used for categorical measures) tested the significance of differences between the intervention and comparison areas adjusting for baseline differences, socio-demographic characteristics and potential confounders. Pre-post changes in the cohort were examined with paired t-tests for continuous variables and McNemar's test for categorical measures.

#### Study 2: Bike count monitoring

1. Is there a significant overall increase in the daily means of bike counts along the cycleway not explained by seasonal, weekend and weather variations?

### Data collection

Four 'Trafficorders', devices that are designed to monitor traffic volumes by type and speed with a reliability range between 95%-98%, were placed at different points along each of the bicycle paths. The devices recorded activity continuously for every quarter of an hour, hourly, and 24 hours for each day during the monitored period. The data were retrieved from the devices as Excel files, separately for each location, and contained all the segmented readings for each day. The 24 hours readings for each location were plotted by dates to check for outliers and to observe time patterns. In addition, precipitation level and the minimum or maximum temperature for each day during the monitored period were provided by the nearest meteorology stations and were included in the data sets. These data were compared over the 24 months of the project.

### Statistical analysis

Negative binomial regression analysis (STATA command 'nbreg') compared the area daily bicycle counts between the intervention and comparison areas over time (using an interaction term) and tested for statistical differences. Negative binomial regression is a regression technique used for nonnegative count variables where the count variation is expected to be greater than that of a true Poisson. The average daily means and the variance over the project period were also calculated for each location and for the intervention and comparison areas as totals.

## Results

### Telephone surveys

A total of 1450 interviews were completed, with a response rate of 64.7 per cent. There was little difference between the intervention and comparison areas in terms of basic demographics at baseline, although there was a higher level of cycling in the intervention area (25% had cycled in the past 12 months compared with 19% in the comparison area). Most respondents (n = 1,254, 86.5%) agreed to be re-contacted 24 months later and to be asked similar questions.

At baseline there was higher bicycle ownership in the intervention area (p = 0.02) (excluding those with a disability), greater use of bicycle paths in the intervention area (p < 0.01) and a slight tendency for respondents in the intervention area to have cycled more recently (data not shown). There were no differences in self-reported health, physical activity levels, minutes riding a bicycle in the past week, and whether respondents had seen any advertising about cycling.

Of the 1,254 respondents at baseline who agreed to be re-contacted, 80.8% (n = 1,013) were able to be contacted, of which 909 agreed to be interviewed (89.7% response rate).

There was a greater proportion of older respondents in the comparison area at the follow-up survey (see Table 2), but otherwise no difference between areas. There was a loss of younger people at the follow-up, as well as students and respondents not born in Australia.

**Table 2:** Demographic characteristics of the baseline sample and study cohort by intervention and comparison areas, and those lost to follow-up

	Baseline (n = 1140)			Cohort (n = 909)			Lost to follow-up (n = 541)
Characteristic	Intervention	Comparison	Total	Intervention	Comparison	Total	Total
	%	%	%	%	%	%	%
SEX							
Male	40.2	41.9	40.9	39.8	39.9	39.8	42.7
Female	59.9	58.1	59.1	60.2	60.2	60.2	57.3
							
AGE							
18-29	17.2	16.5	16.9	14.4	12.7	13.7	22.3*
30-44	33.2	26.9	30.5	32.5	26.1	29.8	31.6
45-60	27.2	25.1	26.3	29.0	24.3	27.0	25.1
61+	22.4	31.5	26.4	24.0	37.0*	29.6	21.0*
							
EDUCATION							
No formal	8.8	8.0	8.4	7.9	7.2	7.6	9.9
School Certificate	24.1	19.8	22.3	25.4	19.3	22.8	21.4
HSC	18.3	17.4	17.9	17.9	16.2	17.2	19.1
Trade	26.3	22.0	24.5	26.2	24.9	25.6	22.5
University	25.9	16.9	20.8	17.7	26.0	21.2	20.1
Other	4.8	6.2	5.4	5.0	6.5	5.6	6.7
							
CURRENTLY STUDYING							
Yes	13.3	14.8	13.9	11.0	13.1	11.9	17.4*
							
COUNTRY OF BIRTH							
Australia	47.3	43.2	45.5	55.4	61.4	58.0	48.5*
							
EMPLOYMENT							
Full-time	39.1	32.1	36.1	39.7	29.3	35.2	37.5
Part-time	11.7	11.6	11.7	12.5	14.4	13.3	8.8
Keeping house	11.6	11.7	11.7	11.4	9.3	10.5	13.7
Aged pension	11.4	11.9	11.6	12.7	12.6	12.7	9.8
Other	26.2	32.7	28.9	23.7	34.4	28.3	30.2

* p < 0.05

At follow-up, almost a quarter (25.8%) of respondents in the intervention group had cycled in the last year compare with 19.4% of respondents cycling in the last year in the comparison area (p = 0.06) (see Table 3). However, this difference is largely explained by the higher level of cycling in the intervention area at baseline (25.2%) compared with the control area (19.3%).

**Table 3:** Cycling uptake in the intervention and comparison areas at the baseline and follow-up survey (n = 909)

	Baseline			Follow-up		
Characteristic	Intervention (n = 520)	Comparison (n = 389)	Total	Intervention (n = 520)	Comparison (n = 389)	Total
	%	%	%	%	%	%
HAS A BICYCLE TO USE						
Yes	32.7	25.4	29.6	44.2*	32.1	39.1
						
RIDER STATUS						
Rode today	0.9	0.6	0.8	1.5	0.8	1.2
Last week	4.7	2.8	3.9	4.4	4.9	6.6
Last month	5.6	5.6	5.6	6.7	3.9	5.5
Last year	13.3	8.7	11.4	12.1	10.0	11.1
Longer than a year	65.0	67.5	66.1	62.5	64.3	63.3
Never	10.4	14.9	12.3	12.7	16.2	14.2
						
Cycled in last year	25.8	19.4		25.2	19.3	
						
PHYSICALLY ACTIVE						
Yes	44.9	47.7	46.1	48.7	53.7	50.8
						
SELF-RATE HEALTH						
Excellent	13.3	16.5	14.6	11.4	12.9	12.0
Good	52.7	49.1	51.2	48.9	50.8	49.7
Fair	27.7	24.7	26.4	30.4	28.9	29.7
Poor	6.4	9.8	7.8	9.4	7.5	8.6
						
SEEN ADVERTISING ABOUT CYCLING						
Yes	12.8	14.4	13.5	17.5	14.9	16.4
						
USED CYCLE PATH						
Yes	22.9 *	15.9	19.9	28.3*	16.2	23.1
						
WANTS TO RIDE MORE						
Yes	69.6	65.1	67.6	62.4*	55.6	59.6

* p < 0.05

At follow-up, there were no differences between the intervention and comparison areas in the proportion of respondents who had cycled in the past year overall (see Table 3) or when the data were stratified by age and sex sub-groups. When type of rider was examined, there were significantly more people who described themselves as novice or beginner riders who had ridden in the past year in the intervention area (11.5%) compared with 1.4% in the comparison area (p = 0.013).

Despite similar path use at baseline, there was a significantly greater use of the bicycle paths in the intervention area (28.3%) at follow-up compared with the comparison area (16.2%) (p < 0.001) (see Table 4) and path use was significantly associated with an almost ten per cent increase in having cycled in the past year (29.1% in the intervention area compared with 20.6% in the comparison area (p = 0.010) (data not shown). There was also a significantly greater proportion of respondents in the intervention area who were likely to use the paths in the future (28.6%) compared with the comparison area (17.8%) (p < 0.001).

**Table 4:** Exposure to the Cycling Connecting Communities and use of bicycle paths by intervention area at follow-up (n = 909)

	Control (n = 389)		Intervention(n = 520)	
	Number	%	Number	%
Seen any cycling ads in last month	58	14.9	91	17.5
Ever heard of CCC	31	8.0	70	13.5*
Participated in any rides or events	8	2.2	12	2.4
Noticed increases in cycling among friends and family	83	21.3	130	25.0
Talked about cycling with friends and family	157	40.4	229	44.0
Has anyone encouraged you to ride	79	21.4	114	22.8
Have you encouraged anyone to ride	121	31.1	182	35.0
Used any of the bicycle paths for any reason	63	16.2	147	28.3**
Likely to use paths in future	63	17.8	140	28.6**

* P < 0.05. ** P < 0.01

A greater proportion of respondents (13.5%) in the intervention area had heard of the Cycling Connecting Communities project compared with the comparison area (8.0%) (p = 0.013) (see Table 4). Among those people who had heard of the CCC project, there was a significantly higher proportion of respondents who had ridden in the last year in the intervention area (32.9%) compared with the comparison area (9.7%) (p = 0.014). This relationship remained significant after adjusting for baseline cycling (p = 0.021). There were no differences by age or sex in the profile of those respondents who recalled awareness of the CCC project, although respondents who described themselves as occasional riders at baseline in the intervention area were most likely to recall awareness of the CCC project (73.7%) compared with the comparison area (23.5%) (p = 0.004). Path use in the intervention area was greater than in the comparison area (p < 0.001) after adjusting for baseline differences, highlighting a greater increase in path use in the intervention area.

### Minutes riding in the last week

In the intervention area, among those that had ridden in the past week there was a slight decrease in the mean minutes cycling for recreation or exercise (169.5 minutes to 152.1 minutes per week), but a large increase in the mean minutes cycling for transport (76.9 minutes to 174.2 minutes per week). In the comparison area there was a much bigger drop in the mean minutes of recreational cycling (190.3 minutes to 121.3 minutes per week) and a large drop in mean minutes of cycling for transport (197.6 minutes to 71.7 minutes per week).

For the small subset of respondents that had ridden in the previous week at both baseline and follow-up (n = 18) a similar pattern was observed (see Table 5).

**Table 5:** Mean minutes cycled and mean number of sessions cycled in the past week (paired data only n = 18)

	Comparison area		Intervention area	
Cycling for exercise	Minutes (n = 6)	Frequency (n = 6)	Minutes (n = 12)	Frequency (n = 12)
Pre	188.3	2.7	120	1.67
Post	133.3	2.0	230	3.0
Difference, t-test	55, p = 0.499	-0.67, p = 0.175	110, p = 0.082	1.33, p = 0.059
				
Cycling for travel				
Pre	85	1.5	35	1.0
Post	6.7	0.667	150	2.33
Difference, t-test	-78.3, p = 0.220	-0.83, p = 0.383	115, p = 0.062	1.3, p = 0.043
				
All cycling				
Pre	273.3	4.17	155	2.67
Post	140	2.67	380	5.3
Difference, t-test	-133.3, p = 0.231	-1.5, p = 0.137	225, p = .021	2.67, p = 0.004

Overall, among those that had ridden in the past week at baseline or follow-up, there was an increase in the total mean minutes cycled in the past week from 188.6 minutes to 233.0 minutes in the intervention area, compared with a decrease in the comparison area from 274.3 minutes to 134.1 minutes. Using the small subset of paired data (riding in past week at both baseline and follow-up), after adjusting for baseline levels of minutes riding, there was a significant increase in the total mean number of minutes riding in the intervention area compared with the comparison area (p = 0.039).

The increase in minutes riding can be explained in part because of an increase in the number of times participants went riding in the past week in the intervention area (2.9 to 4.8 times), and a slight decrease in the comparison area (4.6 to 4.5).

There was no significant difference between the intervention and comparison area with regard to the total mean minutes of physical activity. There was a similar amount of change in the mean minutes of physical activity - from 234.1 to 260.7 minutes per week in the comparison area, and 210.9 to 242.2 minutes per week in the intervention area. Mean minutes of cycling in the past week was significantly associated with total mean minutes of physical activity per week (p < 0.001), after adjusting for area of intervention, age and sex.

There was no statistical difference between the intervention area (48.7%) and the comparison area (53.7%) (p = 0.130) in the proportion of respondents meeting physical activity guidelines of 150 minutes of moderate intensity physical activity per week. However, of those people who met the physical activity guidelines, 28.1% had cycled in the past year (16.0% in the past month) compared with 16.8% of those not meeting the guidelines having cycled (6.5% in the past month) (p < 0.001 for both past year and past month comparisons). Forty per cent of people riding in the past week achieved the recommended minimum physical activity level just by cycling.

### Bicycle count monitoring

**Figure 2:**
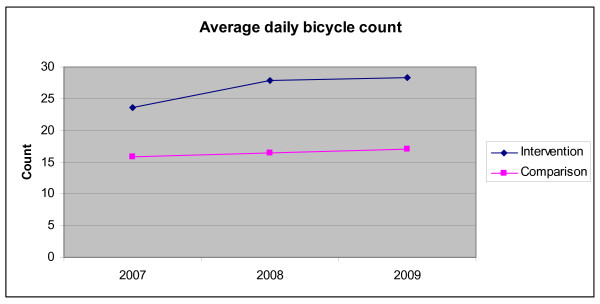
**Bicycle counts in the intervention and comparison areas over time**.

Bicycle count data indicate increases in both the comparison and intervention area, with a significantly greater increase in the intervention area from 23.6 per day (95% confidence interval 21.9 - 25.4) in the first year of the project and which was maintained at the end of the project with 28.3 bicycles counted per day (95% confidence interval 25.6 - 31.1). This represents a 19.9% increase in the intervention area, and is compared with a 12.0% increase in the comparison area. Figure 2 shows the average daily bicycle count by intervention area over time (using westward data).

These results are confirmed in the multivariate analyses (using negative binomial regression and adjusting for weekends, rainfall, minimum and maximum temperatures) with the interaction between area of intervention and time being statistically significant (p = 0.021).

## Discussion

In the intervention area the *Cycling Connecting Communities *project appears to have increased awareness of the project, increased use of bicycle paths, increased cycling among novice or beginner riders, and increased the mean number of minutes cycled in the past week among participants riding at both baseline and follow-up. However, there was no overall increase in the population frequency of cycling, or overall increase in physical activity levels.

The increased use of bicycle paths in the intervention area may have resulted from increased awareness of the network of cycling paths through distribution of project resources such as the new bicycle map (*Discover Fairfield and Liverpool by Bicycle*). As there was no overall increase in the frequency of cycling, it is likely that the project redirected existing cyclists to bicycle paths. The bicycle paths (in both the intervention and comparison areas), while relatively new, already had one in five respondents using them. This level of use indicates that they were not really new facilities.

The stable level of cycling in the intervention areas may represent a positive achievement given the generally declining levels of cycling (8.6% decrease from 1996 to 2006) in the outer areas of Sydney [14,23]. Previous monitoring of travel modes for the journey to work using Australian Bureau of Statistics Census data indicate that there was a relative decline of 27% in bicycle trip mode share in Liverpool from 1996 to 2001 (10% decline in Fairfield) [23]. There were further declines in Liverpool (13%) from 2001 to 2006 while the Fairfield bicycle mode share for the journey to work increased 11% back to 1996 levels [14].

Among those people who had cycled in the past week, there was an increase in the mean number of minutes cycling in the intervention area, with those people using the bike paths and cycling more therefore gaining a health benefit. It is possible that an increase in the overall community prevalence of cycling would lead to an overall increase in population physical activity [24], but this conclusion cannot be reached in this study. Cycling was a significant component of their total minutes of weekly physical activity for those people who cycled, with 40% of cyclists achieving all the minimum 150 hours of moderate intensity physical activity just from cycling. However, there were not sufficient respondents cycling in the past week to influence the overall levels of physical activity.

A US study found that sixty per cent of the cyclists surveyed rode for more than 150 minutes per week during the study and nearly all of the cycling was for utilitarian purposes, not exercise [25]. A disproportionate share of this cycling occurred on streets with bicycle lanes, separate paths, or bicycle boulevards.^25 ^Other research from the US has found positive associations between miles of bicycle pathways per 100, 000 residents and the percentage of commuters using bicycles [26], and that new bicycle lanes in large cities will be used by commuters [27].

Being aware of the CCC project was also associated with a higher frequency of cycling in the intervention area, but the relatively low recall of the project in the community would have minimised possible impacts. A much stronger communication strategy is needed to have an impact at a community level. The overall budget for this project was about $300,000 (AUS) over three years, with the pre- and post- evaluation telephone surveys costing a third of the budget. Crudely, this represents about 35c (AUS) per person per year. This meant there were limited funds for the communication strategy, which had to rely on editorial stories in local newspapers, advertising, letterboxing, and other forms of distributing written information. By comparison, demonstration cycling towns as part of the Cycling England project, received funding of €500,000 per year (approximately €5 per head of population per year), starting in October 2005, and matched by the respective local authorities so that the total level of investment in cycling was at least €10 per head per year (equal to about $25 AUD) [28]. These funds were spent on a mix of infrastructure and behavioural programs. While there is reasonable evidence that the individual project strategies are effective in increasing cycling, the limited project resources meant that only a relatively small proportion of the population were exposed to or participated in project activities. Early results from the Cycling England project indicate increases in cycling and increases in population levels of physical activity [28].

It was disappointing that there was no overall increase in the frequency of cycling in the intervention area. Possible explanations were low levels of exposure to the project and its activities, and long distances to destinations of interest (identified in the baseline survey as a barrier) [29]. Use of higher exposure media such as television or radio may be necessary to achieve adequate dissemination of the message, but this will make the definition of comparison areas more important. It is also possible that a longer period of time is needed to allow for diffusion of innovations to translate into new behaviours.

At baseline, there was an association between cycling in the past year and being sufficiently physically active for men, but not for women. This is consistent with other health survey research that found that men who cycled to work, but not women, were less likely to be overweight or obese compared with other journey to work modes [4,30]. Cycling to work for weight loss or management could be a marketing angle, if it were perceived to be safe.

At baseline the factor most predictive of cycling in the past year was perceived ease of cycling in the respondent's neighbourhood [29]. Having good cycling infrastructure will obviously increase the perception that cycling is easy. Being close to destinations was another significant factor associated with recent cycling [29].

This study highlights that in this outer western Sydney intervention area, which is heavily car dependent, a shift to cycling will require a change in urban planning and density (making destinations of interest much closer), and greater investment in cycling infrastructure where riders want to go, behavioural programs and social marketing. It would be important to repeat this study in a more densely populated urban area, where trip distances were not so great a barrier.

This project raises some questions about the value of limited local social marketing. Policy changes that make car use less appealing (eg increased costs of fuel, less parking availability) are likely to have as much, if not more, impact as information and persuasion campaigns. If only a small amount of resources are available, then maps and bicycle path signage may be a better investment than other forms of communication. Alternatively, targeting a more narrowly defined target group might achieve better results within that sub-population.

The bike count data confirmed the self-reported use of the bicycle paths in the intervention area, confirming the lack of change in the frequency of cycling before and after the intervention. Limitations of these counters were that they were prone to damage and took some time to be repaired, and that they were only in two specific locations in the intervention.

A limitation of the evaluation was that the actual number of people who had cycled in the past week, month or even past year, was relatively low. This meant that statistical power to compare the intervention area with the comparison area was weak. A much larger sample was needed. However, a strength of this project has been the high degree of rigour involved in conducting the pre- and post- evaluation with a control group, with excellent response rates for both surveys, and a high quality data-set provided to the investigators for analysis. The use of bike counters to cross-calibrate the self-reported data is also a strength of the study.

## Conclusions

This study shows that use of cycling infrastructure can be increased with a combination of social marketing and opportunities for people to ride in a safe and social context. Communication strategies that inform potential users of where the infrastructure is located (such as maps and route signposting) are critical. Users of this infrastructure are likely to be existing cyclists and novice or beginning riders who are trialling a new behaviour. Those people who use the cycling infrastructure will tend to cycle for longer if encouraged to ride. However, without sufficient resources, the effectiveness of a community based intervention in increasing population cycling and physical activity is limited.

## Competing interests

The authors declare that they have no competing interests in this study.

## Authors' contributions

CR conceived the idea of this study and undertook data analysis and interpretation and wrote the original draft. CN collected process data and contributed to writing. LMW, DM, JG and AB contributed to the evaluation design, and writing this manuscript. All authors have read and approved the final manuscript.
